# The Process of Acclimation to Chronic Hypoxia Leads to Submandibular Gland and Periodontal Alterations: An Insight on the Role of Inflammatory Mediators

**DOI:** 10.1155/2018/6794508

**Published:** 2018-12-09

**Authors:** Antonela Romina Terrizzi, María Inés Conti, María Pilar Martínez, Javier Fernández-Solari

**Affiliations:** ^1^Cátedra de Fisiología, Facultad de Odontología, Universidad de Buenos Aires, Buenos Aires 1122, Argentina; ^2^CONICET, Consejo Nacional de Investigaciones Científicas y Técnicas, Buenos Aires 1122, Argentina

## Abstract

The exposition to hypoxia is a stressful stimulus, and the organism develops acclimation mechanisms to ensure homeostasis, but if this fails, it leads to the development of pathological processes. Considering the large number of people under hypoxic conditions, it is of utmost importance to study the mechanisms implicated in hypoxic acclimation in oral tissues and the possible alteration of some important inflammatory markers that regulate salivary and periodontal function. It is the aim of the present study to analyze submandibular (SMG) and periodontal status of animals chronically exposed to continuous (CCH) or intermittent (CIH) hypoxia in order to elucidate the underlying molecular mechanisms that may lead to hypoxic acclimation. Adult Wistar rats were exposed to CCH or CIH simulating 4200 meters of altitude during 90 days. Salivary secretion was decreased in animals exposed to hypoxia, being lower in CIH, together with increased prostaglandin E_2_ (PGE_2_) content, TBARS concentration, and the presence of apoptotic nuclei and irregular secretion granules in SMG. AQP-5 mRNA levels decreased in both hypoxic groups. Only the CCH group showed higher HIF-1*α* staining, while CIH alone exhibited interradicular bone loss and increased concentration of the bone resorption marker CTX-I. In summary, animals exposed to CIH show a worse salivary secretion rate, which related with higher levels of PGE_2_, suggesting a negative role of this inflammatory mediator during hypoxia acclimation. We link the weak immunorreactivity of HIF-1*α* in CIH with improper hypoxia acclimation, which is necessary to sustaining SMG physiology under this environmental condition. The alveolar bone loss observed in CIH rats could be due mainly to a direct effect of PGE_2_, as suggested by its higher content in gingival tissue, but also to the indirect effect of hyposalivation. This study may eventually contribute to finding therapeutics to treat the decreased salivary flow, improving in that way oral health.

## 1. Introduction

Saliva is a mixed fluid that derives predominantly from 3 pairs of major salivary glands: the submandibular (SMG), the parotid, and the sublingual glands, producing 70, 20, and 5% of whole saliva, respectively [1, 2]. The secretion of saliva is controlled by the autonomic nervous system. The parasympathetic nervous system is the main controller of this secretion via impulses in the chorda tympani nerve that innervate it and release acetylcholine, which evokes copious salivary secretion by activating muscarinic receptors. The sympathetic nervous system controls salivary secretion by also acting on *α*- and *β*-adrenergic receptors, inducing less volume of thicker saliva. Although the salivary flow is regulated by autonomic reflexes, there are other substances that play an important role in the salivary secretion rate. An increased level of prostaglandin E_2_ (PGE_2_) has been associated with decreased salivary output whereas nitric oxide (NO) is considered to be a potent stimulator of salivary secretion [3]. Saliva represents the first barrier to the entry of bacteria and viruses into the body, and thus, changes in its secretion are important in the statement and progression of oral infectious processes [4]. Diminished salivary output is called “hyposalivation” and significantly affects the individual's quality of life as well as oral health [1, 5] as mechanical cleansing, lubrication, tooth mineralization, and antimicrobial activity are affected. Besides these functions, salivary glands are known to act as immunomodulatory organs that regulate immune/inflammatory reactions within the oral environment [6].

Hypoxemic hypoxia can be defined as a condition wherein the oxygen pressure in the blood is too low to saturate hemoglobin. This type of hypoxia is due to two mechanisms: (1) a decrease in the amount of breathable oxygen due to reduced barometric pressure [7], often encountered in pilots, mountain climbers, and people living at high altitude, and (2) cardiopulmonary failure in which the lungs are unable to efficiently transfer oxygen from the alveoli to the blood. In the case of people inhabiting at high altitude, they are considered to be exposed to chronic continuous hypoxia (CCH) and they represent 2% of the world's population [8]. However, there is another frequent condition of exposition to hypoxia that is not continuous (mountain sports, laboral activities, and diseases such as sleep apnea), known as intermittent hypoxia [9]. The exposition to hypoxia is considered to be a stressful stimulus, and the organism has to develop physiological compensatory mechanisms to ensure homeostasis. Acclimation is the most prevalent phenotypic modification that takes place under hypoxia and involves hematological, cardiovascular, renal, and metabolic changes to help the organism to cope with lower O_2_ levels [10]. At a molecular level, hypoxia evokes highly coordinated cellular responses in order to preserve cell viability. These adaptive responses are orchestrated by the hypoxia-inducible factors (HIFs), “master” regulators of the hypoxic response [11]. The mechanisms that take place under CCH conditions are different compared to intermittent exposure, mainly because of the distinct signaling pathways activated due to the length and intervals of lower O_2_ partial pressure [9]. Despite that the adaptive response is aimed at helping the organism to cope with low oxygenation, hypoxia could also lead to the development of pathological processes if the ability to maintain O_2_ homeostasis fails [12]. It has been accepted that hypoxia is able to induce inflammation. Hypoxia amplifies several molecular pathways that are involved in phagocytosis, leukocyte recruitment, and adaptive immunity, meaning that the activation of HIF-1*α* is necessary to eliminate pathogens [13]. Besides, hypoxia exposure is known to increase cellular oxidative stress leading to the production of reactive oxygen species (ROS) with deleterious effects on lipids, proteins, and DNA [14].

Despite the fact that the effects of hypoxia in the organism are well established, its role on oral health is still not clear. In the salivary gland research field, hypoxia has been shown to decrease salivary secretion in submandibular glands [15]. The mechanisms by which it may induce hyposalivation are still unclear, as the role of HIF-1*α* in acinar cells has not been yet elucidated. Sugimoto et al. [16] reported that aquaporins (AQP), proteins that control water secretion and play an important role during the first step of saliva synthesis, were regulated by HIF, suggesting a possible mechanism by which hypoxia could decrease the salivary flow rate. As mentioned previously, an imbalance in NO or PGE_2_ production could also be linked with decreased saliva volume.

On the other hand, the periodontal field is the one that has received major contributions on the topic. It is known that hypoxic exposure increases alveolar bone loss in rats both with and without experimental periodontitis [17], as well as the level of some important inflammatory mediators during periodontitis pathogenesis, such as NO and tumor necrosis factor *α* [17–19]. However, there is no study that compares alveolar bone loss under the two different types of hypoxic exposition.

The limited O_2_ attained during exposure to chronic hypoxia acts as a challenging stimulus and, considering the large number of people under these circumstances, together with the possible alteration of some important inflammatory markers that regulate salivary and periodontal function under hypoxia, makes it of utmost importance to study the mechanisms implicated in acclimation in oral tissues. Therefore, the aim of the present study was to evaluate the salivary gland and alveolar bone status of rats submitted to chronic continuous hypoxia in order to elucidate the underlying molecular mechanisms that may lead to hypoxic acclimation.

## 2. Methods

### 2.1. Experimental Design

Adult female Wistar rats (initial weight 250 g) were used throughout the experiments. They were weighted and randomly divided into 3 groups of 12 animals each as follows: control (C; normoxic environment), chronic continuous hypoxia (CCH; continuously exposed to 600 mbar, which equals 4200 m of altitude above sea level, placing the animals into a simulated high-altitude chamber 24 h/7 days a week), and chronic intermittent hypoxia (CIH; exposed to 18 h/5 days a week to 600 mbar). These hypoxic schemes were chosen due to the fact that intermittent exposed animals are submitted to the hypoxic stress half the time than the continuous exposed animals, as suggested by other authors in the literature [9, 20]. All animals were allowed free access to water and a standard pelleted chow diet and were treated in accordance with the National Institutes of Health guidelines for the care and use of laboratory animals (NIH 8th edition, 2011). Protocols were approved by the Ethical Commission of the Faculty of Dentistry, University of Buenos Aires (no. 11/06/2012-23). At the end of the experimental period (3 months), animals were euthanized by CO_2_ and weighed. Both hemimandibles and SMG were collected to perform biochemical and histological determinations. Blood samples were taken by cardiac puncture to verify the hypoxic state assessing the hematocrit (%) by micromethod [21].

### 2.2. Salivary Secretion Rate

Two weeks before the autopsy, animals were anesthetized with 2% of xylazine chloride (5 mg/kg; i.p.) and 5% ketamine chloride (50 mg/kg, i.p.) to assess salivary secretion by collecting and weighing salivary samples after stimulation with pilocarpine (0.5 mg/kg, in saline). Briefly, a cotton ball of 20 mg was placed inside the rat's cheek to collect saliva and weighed at the end of 30, 60, and 90 minutes, obtaining 3 measurements of each animal at 3 different time points [22].

### 2.3. Inflammatory Mediators in SMG and Gingival Tissue

#### 2.3.1. Radioimmunoassay (RIA) of PGE_2_

To evaluate PGE_2_ content, SMG or gingiva was homogenized in ice-cold ethanol (100%) and centrifuged at 10,000 x g for 15 min at 4°C, and the supernatant was collected and evaporated in a SpeedVac. The residues were resuspended with RIA buffer, and the Sigma antiserum was used. The PGE_2_ content was expressed as pg/mg of weight tissue [23].

#### 2.3.2. Measurement of Inducible Nitric Oxide Synthase (iNOS) Activity

The activity of iNOS was measured by modifying the method of Bredt & Snyder [24]. SMG was homogenized separately in 500 *μ*l of ice-cold 20 mM HEPES (pH 7.4; Sigma-Aldrich) with EGTA (2 mM) and DL-dithiothreitol (DTT, 1 mM; Sigma-Aldrich). After the tissue was homogenized, NADPH (120 *μ*M; Sigma-Aldrich) and 200,000 dpm of [14C]-arginine monochloride (297 mCi/mmol; Perkin–Elmer, Waltham, MA, USA) were added to each tube and incubated for 10 min at 37°C in a Dubnoff metabolic shaker (50 cycles per min; 95% O_2_/5% CO_2_) at 37°C. The tubes were then centrifuged at 10,000 x g for 10 min at 4°C. The supernatants were applied to individual columns containing 1 ml of Dowex AG 50W-X8 Na^+^ form mesh 200–400 (Bio-Rad Laboratories, Hercules, CA, USA) and washed with 2.5 ml of double-distilled water. All collected effluent fluid from each column was counted for activity of [14C]-citrulline in a liquid scintillation analyser (Tri-Carb 2800TR, Perkin- Elmer). Since NOS converts arginine into equimolar quantities of NO and citrulline, the data were expressed as pmol of NO produced per min per mg of protein [25].

#### 2.3.3. TBARS Content

Thiobarbituric acid-reactive substances (TBARS) were evaluated in SMG quantifying malondialdehyde (MDA) as the product of lipid peroxidation that reacts with trichloroacetic acid-HCl, yielding a pink-stained TBARS determined in a spectrophotometer (Hitachi U-2001) at 540 nm. TBARS were calculated as nanomoles per milligram of tissue [26].

### 2.4. Histological and Immunohistochemical Analyses

#### 2.4.1. Optic Microscopy

A portion of SMG was fixed in a 4% formaldehyde- phosphate saline (PBS) buffer solution, embedded in paraffin, and cut into 5 *μ*m sections to perform routine histologic analyses with hematoxylin and eosin (H&E) staining and immunohistochemistry. After dewaxing and rehydration, slides were submitted to antigen retrieval with citrate buffer pH 6 for 10 minutes in a microwave oven followed by 20 minutes of peroxide blocking reagent exposure (EnVision™ FLEX Systems FLEX, Dako, USA) to block endogenous peroxidase activity. Nonspecific protein binding was blocked using 3% bovine serum albumin (BSA) in PBS. Afterwards, sections were stained with primary antibodies (anti-HIF-1*α* or anti-AQP-5; Biorbyt, UK) at 4°C overnight followed by exposure to horseradish peroxidase-conjugated secondary antibody (EnVision™ FLEX Systems Dako). A brown chromogen was used to detect the primary antibody, and hematoxylin was used as a counterstain [27].

#### 2.4.2. Transmission Electron Microscopy

Ultrastructural analyses of SMG morphology were done by transmission electron microscopy (TEM). Briefly, tissue blocks of approximately 1 mm^3^ were fixed in 1% glutaraldehyde in 0.1 M phosphate buffer (pH 7.4) for 4 hours and then washed 3 times with 0.1 M phosphate buffer. After dehydration and embedding in Durcupan resin (Fluka AG, Switzerland), tissue sections of 5 nm were cut and stained with uranyl and lead citrate. Sections were observed under a Zeiss EM 109T transmission electron microscope.

### 2.5. mRNA Levels of HIF-1*α* and AQP-5 in SMG

Pieces of SMG of approximately 50 mg were homogenized with RNAzol Reagent and stored at −80°C until used. RNA was extracted according to the manufacturer's indications (Molecular Research Center Inc., Cincinnati, OH, USA) and quantified by NanoDrop (Eppendorf, Hamburg, Germany). Afterwards, cDNA was synthesized from total RNA (3 *μ*g) using M-MLV RT, ribonuclease inhibitor, and random primers. The specific primers were designed using Primer 3 Software, and the sequences were *β*-actin: forward 5′ACCCGCCGAGTACAACCTTC 3′ and reverse 5′ATGCCGTGTTCAATGGGGTA 3′ (94°C 5 min; 35 cycles of 94°C 40 s, 58°C 30 s, and 72°C 1 min; and 72°C 5 min) product (bp): 156; AQP-5: forward 5′GAGATTCGTGAATGCGGTGC3′ and reverse 5′GTGGTTTATTGGGAAGCGCC3′ (94°C 5 min; 35 cycles of: 94°C 40 s, 62°C 30 s, and 72°C 1 min; and 72°C 5 min), product (bp) 256; HIF-1*α*: forward 5′TGCTTGGTGCTGATTTGTGA3′ and reverse 5′GGTCAGATGATCAGAGTCCA3′ (94°C 5 min; 35 cycles of 94°C 40 s, 55°C 30 s, and 72°C 1 min; and 72°C 5 min) product (bp) 210. Products were loaded onto 2% agarose gel, and bands were visualized on a transilluminator under UV light. Photographs were taken with a digital camera (Olympus C-5060) and analyzed with the ImageJ software package. The relative mRNA level was normalized to *β*-actin, and results were expressed as arbitrary units (AU) of relative optical density [28].

### 2.6. Alveolar Bone Loss Determinations

#### 2.6.1. Cortical Bone Loss by Distance Method

Hemimandibles were resected, defleshed, and stained with 1% aqueous methylene blue to delineate the cement-enamel junction (CEJ) and the alveolar crest (AC) [29]. A stereomicroscope and a digital caliper were used to measure three buccal and three lingual distances (mesial, central, and distal), from the CEJ to the AC. The sum of the three distances of each side of molars was used as a measure of the alveolar bone loss in millimeters.

#### 2.6.2. Interradicular Bone Loss

Hemimandibles were fixed in formalin buffer. Afterwards, they were decalcified in 10% EDTA pH 7 for 45 days, dehydrated with ethyl alcohol, and clarified with xylene. The sector containing the first molar was embedded in paraffin, and 7 *μ*m sections of each first molar oriented mesiodistally were obtained and stained with H&E. Histomorphometric evaluation was performed on digitalized microphotographs using Image-Pro Plus 4.5 software. Interradicular bone volume was measured as bone volume (BV)/total volume (TV) (%). TV was taken as bone tissue plus bone narrow and periodontal ligament [30]. Histopathological analyzed of the gum tissue around the first molar was also performed.

#### 2.6.3. C-Terminal Telopeptide of Collagen Type I (CTX-I) Concentration

The concentration of this bone resorption marker was determined in serum by using an ELISA commercial kit, following the manufacturer's instructions (Fine Test, Wuhan Fine Biotech Co., Wuhan, China).

## 3. Statistics

Experiments were performed at least three times, and figures represent results of individual experiments. Data were expressed as mean ± SEM. Analysis of variance followed by Tukey's test for multiple comparisons was used to determine statistical significance (*p* < 0.05). Statistical analysis was performed using the software InfoStat (Córdoba, Argentina).

## 4. Results

### 4.1. Hematocrit and Morphometric Measures

At the end of the experimental period, body and SMG weight were determined. These morphometric measures were not statistically different among groups. As all the animals ate the same amount of food, we consider that the changes in the parameters further described in SMG would not be due to nutritional state. To verify the hypoxic state, hematocrit was assessed. Increased values were found in both hypoxic groups compared with control animals, being higher in continuous exposed rats (15% higher in CIH vs. 29% higher in CCH) (Table 1).

### 4.2. Exposition to Chronic Hypoxia Decreases Total Salivary Flow Rate, Mainly during Intermittent Exposure

**Figure 1:**
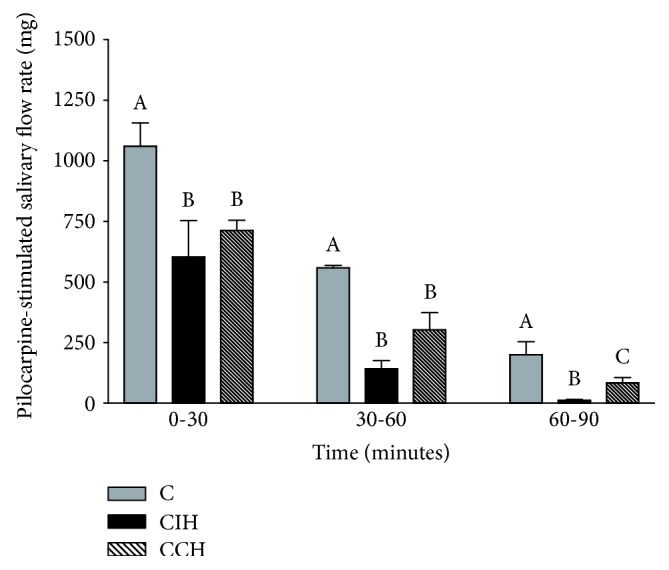
Effect of hypoxia on total salivary response to pilocarpine. C: control; CIH: chronic intermittent hypoxia; CCH: chronic continuous hypoxia. Results are presented in mean ± SEM. Statistics: *a* ≠ *b* ≠ *c*, *p* < 0.05.

In order to explore the effects of hypobaric hypoxia on salivary flow, pilocarpine was used to stimulate salivary secretion. Total collected saliva was decreased in both hypoxic groups within the first 30 min of stimulation compared to C (ANOVA *F* (2, 13) = 4.88, *p* < 0.05). After 60 min, the same pattern was observed, but salivary secretion was lower against C in intermittently exposed animals (75% in CIH; *p* < 0.01 vs. 46% in CCH; ANOVA *F* (2, 13) = 12.47, *p* < 0.05), without statistical difference between both hypoxic animals. After 90 min, total salivary secretion in CIH was 95% lower than C animals and in CCH the amount of saliva was 59% lower than C, with existing statistical difference in both hypoxic groups (ANOVA *F* (2, 12) = 5.62, *p* < 0.05) (Figure 1). In summary, this study reflects a lower salivary secretion rate in animals exposed to hypoxia, with secretion being more impaired during intermittent exposure.

### 4.3. PGE_2_ Content and TBARS Concentrations in SMG Are Enhanced during Chronic Hypoxia

**Figure 2:**
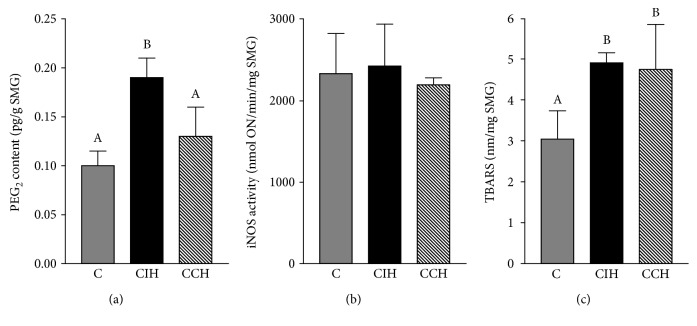
Biochemical determinations in SMG. (a) PGE_2_ content, (b) iNOS Activity, and (c) TBARS concentration. C: control; CIH: chronic intermittent hypoxia; CCH: chronic continuous hypoxia. Results are presented in mean ± SEM. Statistics: *a* ≠ *b* ≠ *c*, *p* < 0.05.

To understand the link between prostaglandins and salivary secretion, PGE_2_ content was measured, as a negative correlation between PGE_2_ content and salivary function has been established [3]. CIH significantly increased PGE_2_ content compared to both control and CCH (Figure 2(a)) (ANOVA, *F* (2, 12) = 6.85, *p* < 0.05). The activity of iNOS was also analyzed, as nitric oxide derived from this enzyme is a potentially significant factor altering salivary secretion in conditions that affect SMG. No statistical difference in iNOS activity was found between control and both hypoxic groups (Figure 2(b)) (ANOVA, *F* (2, 14) = 0.30, *p* > 0.05). Since oxidative stress is known to be a hallmark of hypoxia-mediated tissue damage, TBARS were assessed in SMG. Both types of exposure significantly enhanced TBARS content in SMG (Figure 2(c)), suggesting the existence of lipid peroxidation in the gland (ANOVA, *F* (2, 15) = 5.81, *p* < 0.05).

### 4.4. Intermittent Hypoxia Induces Ultrastructural Alterations in SMG

**Figure 3:**
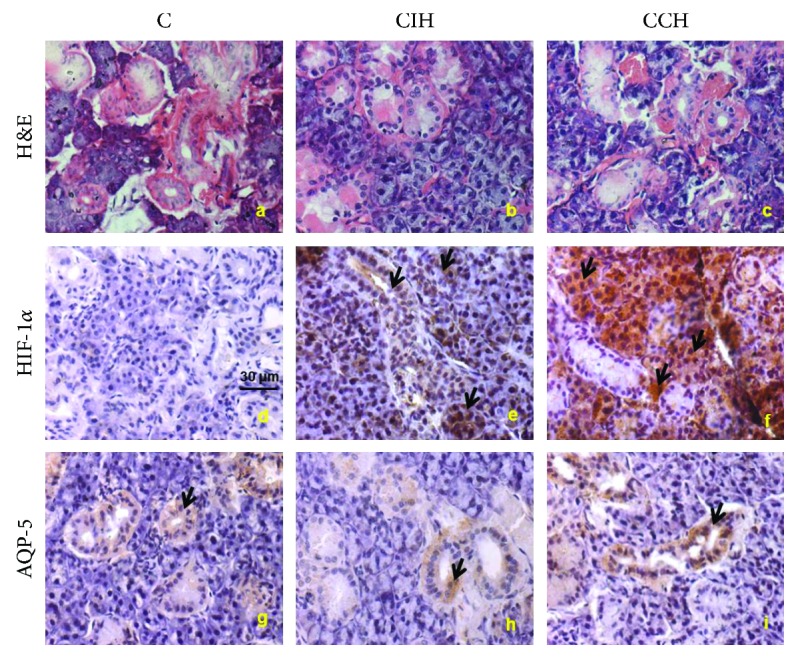
Histological analyses of SMG. Photographs of SMG of one randomly selected slide per group. Resected SMG were observed under a Zeiss Axiophot microscope (40x). Scale bar = 30 *μ*m. C: control; CIH: chronic intermittent hypoxia; CCH: chronic continuous hypoxia. SMG stained with H&E (a, b, and c). HIF-1*α* (d, e, and f) immunohistochemistry. AQ-5 immunoreactivity (g, h, and i) *n* = 6 animals per group. Arrows indicate HIF-1*α* and AQP-5-positive cells.

**Figure 4:**
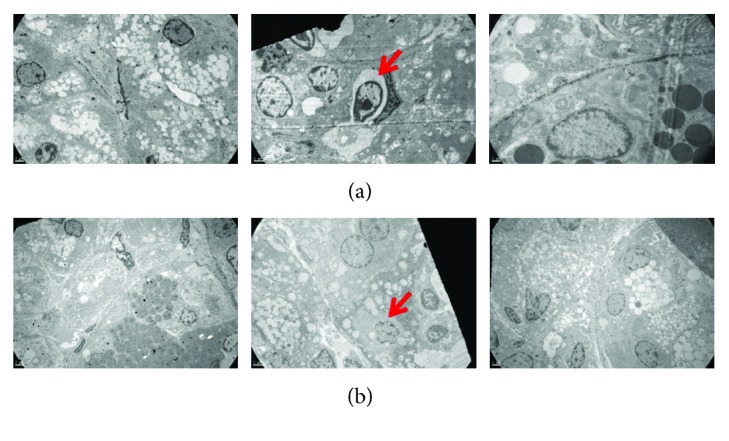
Ultrastructural analyses of SMG. Photographs of SMG of one randomly selected slide per group observed under a Zeiss EM 109T (3000x). Scale bar = 2 *μ*m. C: control; CIH: chronic intermittent hypoxia; CCH: chronic continuous hypoxia. (a) Acini and (b) intercalated ducts. Arrows indicate apoptotic nuclei.

Regarding routine histological analyses in SMG, no parenchymal or stromal changes were observed in any group (Figures 3(a)–3(c)). However, electronic microscopy analyses revealed apoptotic cells in acini and intercalated ducts and irregular and fewer secretory granules in acini of SMG of the CIH-exposed group. Normal epithelial cell morphology was observed in serous and mucous acini of the C and HCC groups (Figures 4(a) and 4(b)).

### 4.5. Higher HIF-1*α* Immunorreactivity Was Observed in SMG of CCH Animals

RT-PCR studies revealed that HIF-1*α* mRNA levels in SMG were higher in both hypoxic groups compared to C (whose level was not detectable), but there were no statistical differences between CIH and CCH (ANOVA *F* (2, 12) = 27.75, *p* < 0.05) (Table 2). When analyzing the localization of HIF-1*α* by immunohistochemistry, we found, as expected, lack of staining in the C group. SMG of animals exposed to hypoxia showed higher immunorreactivity of this transcription factor in the cytoplasm, mainly in serous acini and ducts, with low or no color in mucous acini. SMG of the CIH group showed irregular staining, with areas of no expression similar to the C group. Only the glands of the CCH group showed homogeneous increase in HIF-1*α* immunorreactivity (Figures 3(d)–3(f)).

### 4.6. Hypoxia Diminished AQP-5 mRNA Levels in SMG

It has been shown that alteration in AQP-5 expression may lead to hyposecretion in salivary glands. In our present study, exposition to hypoxia decreased mRNA levels of AQP-5 in SMG of both hypoxic groups, suggesting that this could be a possible explanation for the impaired salivary flow in salivary glands of hypoxic rats (ANOVA *F* (2, 12) = 29.48, *p* < 0.05) (Table 2). However, immunohistochemical analyses of AQP-5 revealed no significant changes in its localization or immunorreactivity due to hypoxia. Staining was localized mainly in intercalated ducts and mucous acini, with no immunorreactivity in serous acini (Figures 3(g)–3(i)).

### 4.7. Intermittent Hypoxia Exposure Increased Cortical and Interradicular Alveolar Bone Loss

**Figure 5:**
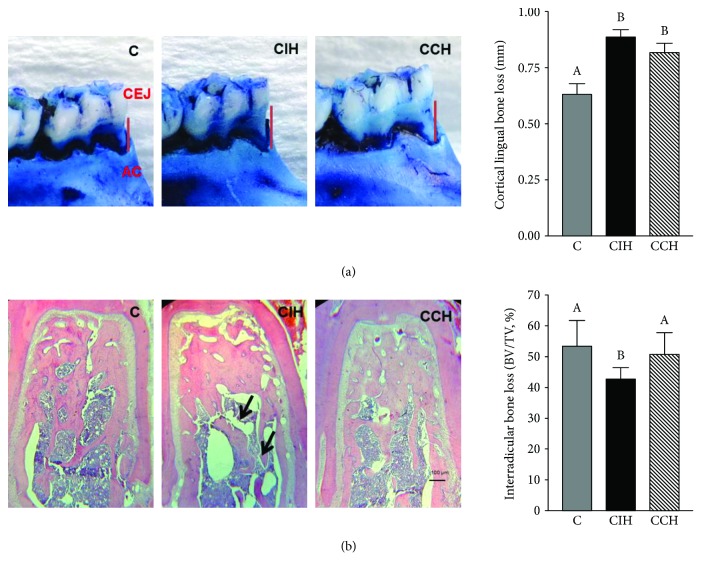
Cortical and interradicular alveolar bone loss assessment. (a) Lingual bone loss (mm). Photographs of the lingual side of the mandible in the lower first molar region of one animal per group selected randomly. (b) Interradicular bone loss (BV/TV, %). Photographs of transverse slides of the longitudinal sections of the mandibular interradicular bone in C (control), CIH (chronic intermittent hypoxia), and CCH (chronic continuous hypoxia) of one animal per group selected randomly (2.5x). Scale bar = 100 *μ*m. Results are presented in mean ± SEM. Statistics: *a* ≠ *b* ≠ *c*, *p* < 0.05.

**Figure 6:**
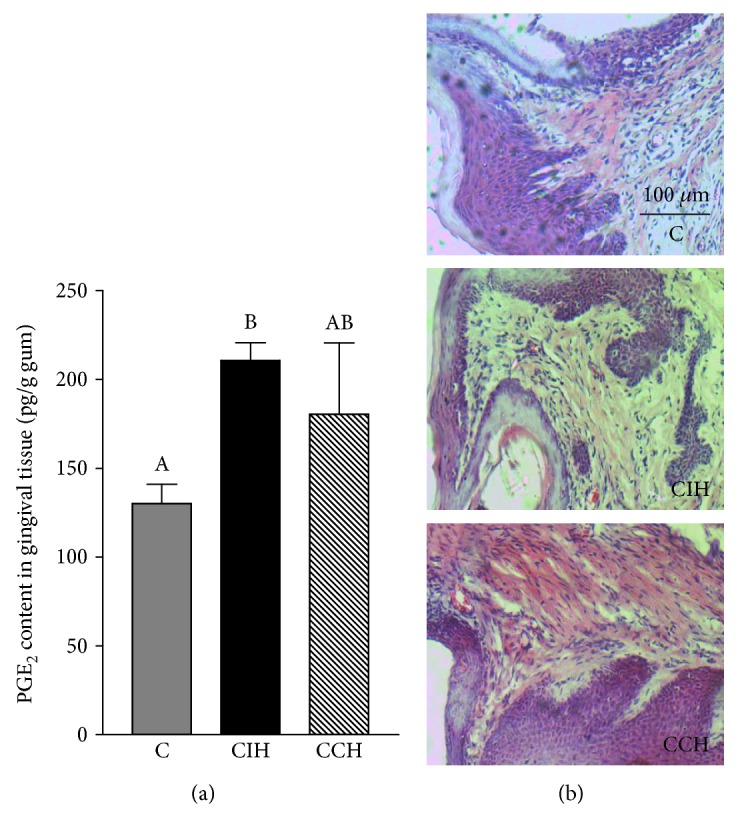
PGE_2_ content and histomorphometrical analyses in gum tissue. (a) PGE_2_ content in gingival tissue of animals exposed to CIH (chronic intermittent hypoxia) and CCH (chronic continuous hypoxia). Results are presented in mean ± SEM. Statistics: *a* ≠ *b* ≠ *c*, *p* < 0.05. (b) Photograph of the gingival tissue surrounding the lower first molar of one animal per group selected randomly (40x). Scale bar = 100 *μ*m.

**Figure 7:**
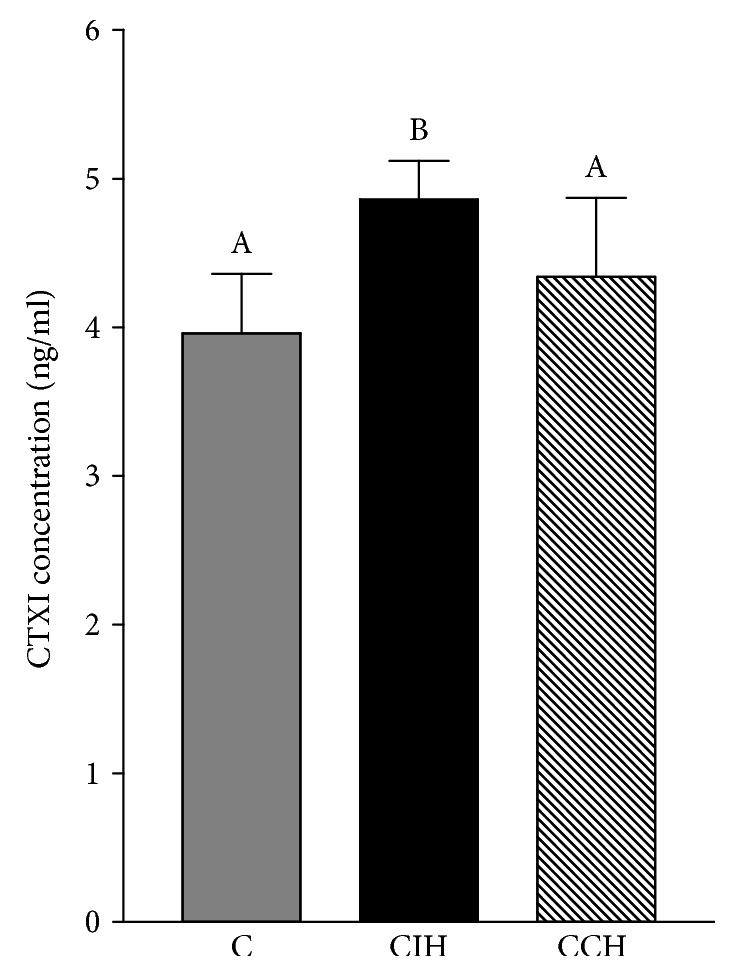
CTX-I concentration in serum. CIH (chronic intermittent hypoxia) and CCH (chronic continuous hypoxia). Results are presented in mean ± SEM. Statistics: *a* ≠ *b* ≠ *c*, *p* < 0.05.

To assess alveolar bone loss, both cortical and interradicular bone was measured. Both hypoxic groups showed increased cortical bone loss at the lingual side of the mandible (ANOVA *F* (2, 15) = 54.50, *p* < 0.05) (Figure 5(a)) as determined by the distance method. When interradicular bone volume was analyzed, we found that only CIH significantly enhanced this parameter (ANOVA *F* (2, 12) = 4.70, *p* < 0.05) and increased bone marrow cavity when compared to C and CCH-exposed animals (Figure 5(b)). A higher content of PGE_2_ was found in gingival tissue surrounding the lower first molar only in CIH (Figure 6(a)). With this molecule being a known stimulator of bone loss during periodontal disease [21], we can link the observed bone loss in intermittent exposed animals to the increased levels of this inflammatory mediator. When analyzing gingival tissue by optic microscopy, we found no evidence of inflammatory response. Both epithelial and connective tissue showed normal structures in gums of the 3 groups (Figure 6(b)). Furthermore, only the CIH group showed increased concentration of CTX-I in serum, indicating increased bone turnover (ANOVA *F* (2, 31) = 12.03, *p* < 0.05) (Figure 7).

## 5. Discussion

Salivary glands are key organs in the regulation of hydric, mineral, and immunologic balance of the oral environment [31]. Besides its mechanic cleansing effect, saliva also exerts a role in mucosal host defense thanks to the presence of secretory immunoglobulin and many antimicrobial proteins. A decrease in salivary flow and/or an alteration in saliva composition lead to bacterial overgrowth and increased inflammatory response, which may contribute to pathological bone resorption and tissue detachment observed in periodontal disease [6]. In this study, we show that exposure to chronic hypoxia impairs salivary gland secretion. Intermittent exposure seems to be more detrimental than continuous exposition, as a 95% decrease in salivary secretion was observed after 90 minutes of pilocarpine stimulation vs. 59% decrease in CCH. Other authors have reported studies dealing with salivary flow in animals or humans submitted to high altitude [15, 32], but none of these studies were performed chronically at altitudes where human life can develop. That is to say, the hypoxic conditions selected for this study that simulate 4200 meters above sea level are the ones in which humans can develop acclimation mechanisms to compensate the low values of O_2_.

To understand the mechanisms of the effect observed in salivary secretion, many factors should be analyzed. First, morphometric differences of the organ among animals of the experimental groups, such as size and weight, should be ruled out. In the model used in this study, both corporal and submandibular gland weights were statistically equal in the three groups. This is related to the fact that adult animals ate the same amount of food in control and experimental conditions, allowing them to grow and develop similarly. Previously reported studies from our laboratory had shown that the development of growing rats was affected by hypoxia [17, 21, 30], the reason why this study was planned with adult animals.

**Figure 8:**
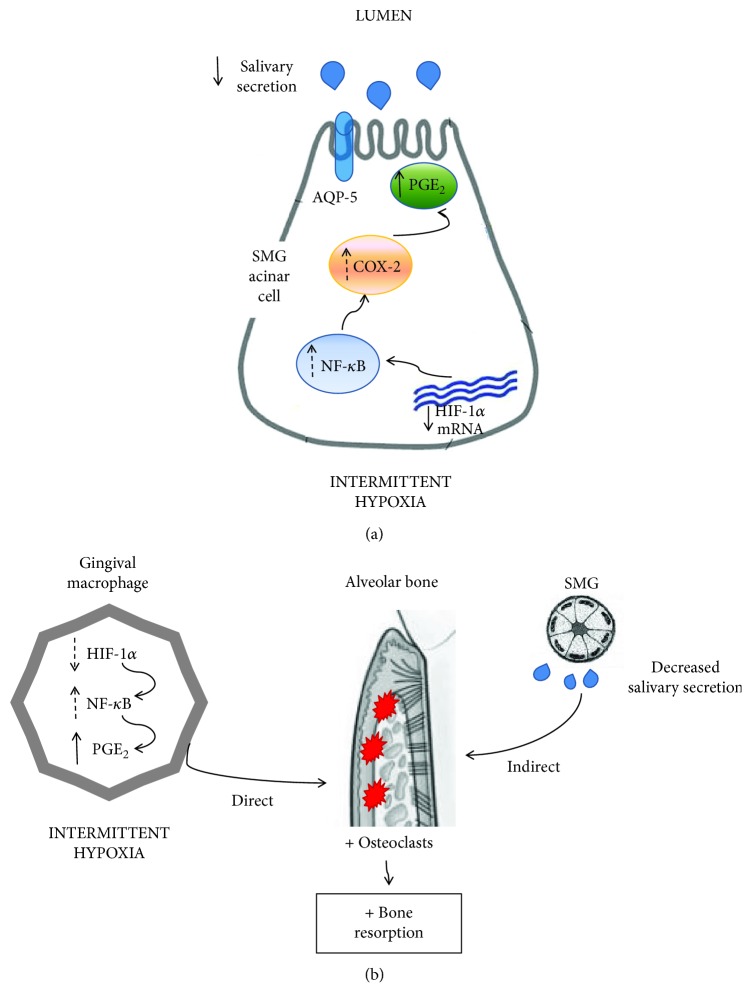
Proposed mechanism that leads to hyposalivation (a) and alveolar bone resorption (b) during exposure to intermittent hypoxia. The schemes contain the molecular mediators analyzed in this study (PGE_2_, HIF-1*α*, and AQP-5: full arrow). However, multiple factors not included in the study participate in salivary secretion and alveolar bone metabolism, such as NF-*κ*B and COX-2 (dotted arrow). We hypothesize that more degradation of HIF-1*α* due to the intermittent normoxic periods in the CIH group would be related with upregulation of NF-*κ*B, leading to higher concentrations of PGE_2_, which is associated with lower levels of saliva and osteoclast genesis and activity. Furthermore, the decreased levels of saliva would constitute an indirect mechanism which increases alveolar bone resorption.

Secondly, prostaglandins in SMG have been reported by other authors as modulatory molecules leading to salivary response inhibition [3, 22]. We observed increased levels of PGE_2_ in SMG of CIH-exposed rats, which would explain the severe hyposalivation observed after 90 minutes of pilocarpine stimulation. This result could be associated with a higher activity of cyclooxygenase-2 (COX-2), which is increased under stressful conditions. Even though both types of hypoxia exposure have been reported to upregulate COX-2 expression, it has been established that longer periods of exposition returned levels of COX-2 to baseline [33–35]. In the case of CIH-exposed animals, the cyclic exposition to hypoxia would increase this enzyme expression compared to the CCH group. Other molecules have been reported to be enhancers of the salivary response, such as nitric oxide produced by iNOS which increases the cholinergic salivary response [36]. In this study, we did not find a higher activity of this enzyme in SMG, suggesting that the concentration of NO derived from iNOS in hypoxic animals does not differ from those under physiological conditions. Increased activity of this enzyme is related with the presence of inflammatory infiltrate within the SMG due to direct aggressions to the organ (i.e., injection with lipopolysaccharide) [36], which is not the case of our experimental model. Besides iNOS, SMG also contains neural NOS and endothelial NOS, so levels of NO deriving from those enzymes should be also considered. Furthermore, higher concentrations of TBARS were found in the SMG of both experimental groups, suggesting that oxidative mechanisms could be associated with the decreased salivary flow observed. Impaired salivary secretion was further evaluated by the analysis of AQP-5. As aquaporins are molecules involved in transcellular water transport in salivary glands, their participation in the secretion rate is essential [37]. Under resting conditions, salivary glands maintain basal saliva secretion, while upon demand upregulation of the secretion is achieved by autonomic innervation. Parasympathetic stimulus through acetylcholine induces translocation of AQP-5 from intracellular vesicles to the apical membrane, allowing their function as water channels [38]. In several pathophysiological conditions, such as Sjögren syndrome, radiation therapy, diabetes, and senescence AQP-5 are reported to be downregulated or with a different localization [37], all of which correlate with reduced salivary secretion. On the other hand, the activation of HIF-1*α* during hypoxia has been associated with the upregulation of aquaporin, leading to a compensatory increase in water transportation in many organs [16, 39]. In the experimental setting of this study, no significant differences regarding AQP-5 distribution or localization were observed in the SMG of any experimental groups. However, the exposition to hypoxia decreased AQP-5 mRNA levels in the gland, suggesting that this molecule expression may be downregulated in this experimental model. In the case of hypoxia, the upregulation of AQP-5 mediated by the parasympathetic nervous system could be affected, as it is known that it induces sympathetic activation [40], leading to lower volumes of a less fluid saliva. Besides AQP-5, other important mediators had been reported to play an important role in the mechanism of salivary secretion. To further analyze the molecular mechanism underlying salivation and hypoxia, HIF-1*α* was assessed in SMG. This transcription factor plays an integral role in the cellular adaptation to low O_2_ concentrations, being essential for immunological responses, vascularization, and anaerobic metabolism [41]. In this study, an increased localization of HIF-1*α* in SMG of animals exposed continuously to hypoxia compared to intermittent exposure was shown. This could be due to the less time of exposure in the CIH group, which would induce more degradation of this transcription factor compared to the animals that spend longer periods under hypoxia (CCH) [42]. The increased catabolism of HIF-1*α* in CIH would mean less transcription of genes needed for the acclimation process, leading to a worse coordinated response to ensure the proper glandular function. It has been established that the activation of HIF-1*α* was related with less inflammatory state mediated by downregulation of NF-*κ*B activation in periapical tissue [43], so we think that the NF-*κ*B molecular pathway could be downregulated in the submandibular gland of CCH animals, enabling a better response to hypoxia (Figure 8). Furthermore, dental stem cells with overexpression of HIF-1*α* had been shown to have a longer lifespan due to NK-lysis resistance [44], which reinforces the concept of this transcription factor being necessary for cellular health and survival. Contrary to our hypothesis, when assessed by PCR, we found similar levels of HIF-1*α* mRNA in SMG of CIH and CCH groups. However, this does not rule out the possibility of less HIF-1*α* in the glands of CIH as posttranscriptional changes on the protein expression and localization could take place. At the histological level, parenchyma and stromal architecture should be taken into consideration to fully evaluate gland morphology. In this study, serous and mucous acini as well as intralobular and secretory ducts showed a normal structure when observed under an optic microscope. Many authors have analyzed submandibular gland histoarchitecture in different animal models where salivary flow was decreased, and none of them have reported changes in the organ assessed by routine histological methods [22, 45]. Nevertheless, ultrastructural mechanisms which cannot be observed by optic microscopy could be playing a role in this experimental model and could explain the alterations in glandular function. In this study, transmission electron microscopy was employed to further analyze SMG morphology. Irregular secretory granules along with some apoptotic nuclei were found in cells of acini and intercalated ducts of CIH animals, whereas these alterations were not observed in the CCH group. These findings could indicate that not only saliva volume but also its quality might be affected under intermittent exposure, as secretory granules contain biologically active molecules that are crucial to ensuring saliva functions.

Regarding periodontal status in hypoxic rats, we found cortical bone loss due to hypoxia only in the lingual side of the mandible, with no effect at the buccal side. Interradicular bone loss was observed only due to CIH exposition, and this correlated with enhanced levels of CTX-I, a marker of collagen degradation due to increased bone resorption. Intermittent exposition to hypoxia would seem responsible for the increase in the content of PGE_2_ in gingival tissue, a potent stimulator of bone resorption, which would constitute a possible molecular mechanism that could explain higher osteoclast activity in this group. Besides this direct effect of hypoxia on osteoclast, it has been demonstrated that low levels of oxygen were capable of suppressing periodontal ligament cell migration and proliferation via the Wnt/*β*-catenin signaling pathway [46], showing the intricate interplay between different periodontal tissues and molecular mechanisms of damage under hypoxic conditions. It is worth mentioning that we did not observe evidence of an ongoing inflammatory response in this experimental model, as evidenced by the absence of inflammatory infiltrate in soft tissue surrounding the teeth. This finding, together with the fact that interradicular bone resorption seemed to occur at the expense of bone marrow expansion, leads us to believe that hypoxia does not induce alveolar bone resorption though the activation of the innate immunity as it would happen in periodontal disease, but the bone resorption observed herein could be associated with an acclimation mechanism where hypoxia stimulates bone marrow expansion, as it happens normally in long bones [47]. The decreased salivary secretion would also contribute as an indirect mechanism with the alveolar bone loss observed under hypoxia, as situations with impaired saliva production had been related with increased values of CEJ-AC distance [6]. This may be explained by the fact that low salivary volume induces higher risk of oral infection due to decreased antimicrobial products, secretory immunoglobulin A, and salivary growth factors.

## 6. Conclusions

The different conditions through which animals are exposed to hypoxic stress appear to differentially activate cellular responses discussed herein. Animals exposed intermittently to hypoxia show worse SMG function than the ones submitted continuously, and this correlated with detrimental periodontal status in CIH. These findings were associated with lower immunorreactivity of HIF-1*α* and higher levels of PGE_2_, both at SMG and gingival levels, which suggest a negative role of this inflammatory mediator during hypoxic acclimation. Besides, our study suggests a distinct phenotypical variation in both experimental conditions that involves ultrastructural changes in SMG. Understanding whether a hierarchy among these two types of hypoxic response mechanisms exists and which are the precise timing and conditions of each mechanism to be activated will improve the knowledge of the biochemical mechanisms underlying hypoxia in oral tissues. This may eventually contribute to shedding light into the processes that regulate salivary gland adaptation and to finding therapeutics to treat the decreased salivary flow, improving in that way oral health.

## Figures and Tables

**Table 1:** Hematocrit and morphometrical measurements. Body weight (g), SMG weight (mg), and hematocrit (%) in control (C), chronic intermittent hypoxia- (CIH-), and chronic continuous hypoxia- (CCH-) exposed animals. Results are expressed as mean ± SEM. Statistics: *a* ≠ *b* ≠ *c*, *p* < 0.05.

	Control	CIH	CCH
Body weight (g)	357.20 ± 31.33^a^	320.41 ± 21.41^a^	337.08 ± 17.60^a^
SMG weight (mg)	218.16 ± 16.40^a^	198.20 ± 10.86^a^	192.87 ± 21.17^a^
Hematocrit (%)	53.10 ± 4.18^a^	61.09 ± 5.74^b^	68.52 ± 5.03^c^

**Table 2:** AQP-5 and HIF-1*α* mRNA expression. Results are expressed as relative optical density in arbitrary units (AU) and normalized to *β*-actin mRNA expression. Statistics: *a* ≠ *b* ≠ *c*, *p* < 0.05. n/d: nondetectable.

mRNA levels in SMG (AU)	Control	CIH	CCH
AQP-5/*β*-actin	5.39 ± 0.89^a^	2.31 ± 0.64^b^	1.95 ± 0.75^b^
HIF-1*α*/*β*-actin	n/d^a^	1.72 ± 0.51^b^	1.31 ± 0.42^b^

## Data Availability

The data used to support the findings of this study are included within the article.
